# A Landscape of *Drosophila melanogaster* Disease Models: From Genetic Platforms to Cross-Disease Mechanisms and Translational Research

**DOI:** 10.3390/biology15171521

**Published:** 2026-09-03

**Authors:** Min-Jin Kwak, Taeyeong Kim, Yebin Yang

**Affiliations:** Department of Forest Products and Biotechnology, Kookmin University, Seoul 02707, Republic of Korea

**Keywords:** *Drosophila*, nervous system disorder, metabolic disease, cancer, infectious disease, gut microbiome, neuromuscular disease

## Abstract

Modeling human diseases using the fruit fly (*Drosophila melanogaster*) has established itself as a cornerstone of functional genomics and preclinical medicine. Despite its anatomical simplicity, the *Drosophila* genome shares remarkable functional conservation with human disease-related genes, enabling the study of complex physiological traits through accessible tissue models. Furthermore, beyond individual disease models, we propose a framework demonstrating how these diseases converge at common molecular centers, such as the breakdown of protein homeostasis, mitochondrial dysfunction, chronic inflammation, and organ-to-organ communication. Finally, we discuss strategies for integrating the *Drosophila* platform into drug development pipelines and establishing standards to enhance inter-laboratory reproducibility. Overall, this review highlights the enduring value of fruit flies as a model system, particularly when combined with AI-omics approaches to transform complex biological datasets into actionable therapeutic strategies.

## Simple Summary

This review highlights that *Drosophila* possesses high functional conservation, serving as a powerful model for human disease research across key fields such as neurodegenerative diseases, metabolic disorders, cancer, immunity, barrier function, and neuromuscular diseases. It examines methods for constructing these models utilizing gene control tools and quantitative analyses and presents a framework for connecting these models through common molecular mechanisms, such as disruption of protein homeostasis, mitochondrial dysfunction, chronic inflammation, and organ-to-organ interactions. Finally, it discusses strategies for integrating *Drosophila* platforms into drug development, enhancing cross-laboratory reproducibility, and combining them with AI-omics to transform complex biological data into targeted therapies.

## 1. Introduction

Understanding complex human diseases requires experimental models that combine clear mechanistic insights with the integration of the whole organism. Over the past few decades, the fruit fly (*Drosophila melanogaster*) has evolved from a tool for basic genetic research into a powerful platform for modeling human pathology [1]. While mammalian models resemble human anatomy, they often have limitations due to genetic redundancy and low experimental throughput [2,3]. In contrast, the fruit fly genome shares functional homologous genes with approximately 75% of human disease-related genes and minimized genetic redundancy [4]. This genetic simplicity and highly versatile tool allow researchers to analyze systemic diseases through individual mechanisms at cellular and tissue levels [5].

A key basis for using *Drosophila* in the study of human diseases is establishing functional equivalence rather than strict anatomical equivalence [6]. While *Drosophila* lacks organs with complex multi-chambered structures, its tissues perform highly conserved physiological roles [7,8]. For example, the *Drosophila* fat body centrally controls metabolic homeostasis, regulates nutrient sensing, energy storage, and systemic endocrine signaling, performing functions like those of the mammalian liver and adipose tissue [9]. Similarly, the *Drosophila* midgut reflects the human digestive tract and contains active intestinal stem cells that regulate epithelial cell regeneration, intestinal barrier integrity, and mucosal immunity [10]. Furthermore, the fundamental mechanisms of synaptic transmission in the central nervous system and the relationship between excitation and contraction in the muscular system are like those in vertebrates, providing a robust basis for modeling neurodegenerative and neuromuscular diseases [11,12,13]. By focusing on these functional similarities, researchers can reliably model long-term specific pathologies and complex inter-tissue communications in a simplified in vivo environment [7,14].

Despite these functional advantages, interpreting *Drosophila* disease models requires a clear understanding of their experimental limitations. Due to their lack of a closed vascular network, complex organ structure, and adaptive immune system, *Drosophila* cannot fully replicate mammalian organ-to-organ interactions or antibody-mediated inflammatory responses [15]. However, this anatomical simplicity can be a significant advantage, as human pathologies stem from the breakdown of core cellular processes [6]. By eliminating confounding physiological variables common in vertebrate systems, *Drosophila* models offer a high signal-to-noise ratio, allowing researchers to isolate interactions between specific pathways and establish clear, directional causal relationships [16]. Collectively, the value of the *Drosophila* platform lies not in faithfully replicating every clinical feature of human disease, but in breaking down complex pathologies into manageable molecular modules that can guide targeted translational studies.

## 2. Experimental Architecture for Disease Modeling

Building disease models in *Drosophila* requires an integrated experimental framework that combines precise genetic engineering with quantitative phenotypic analysis [7,17]. Its primary objective is to reproduce specific pathogenicity mechanisms while strictly controlling environmental and genetic variables [17,18]. This framework is supported by two complementary pillars: genetic tools to isolate phenotypic effects in specific tissues and time windows [19,20] and a standardized evaluation panel to measure the functional impacts of these disturbances [7]. By combining targeted genetic drivers with rigorous phenotypic assays, researchers can establish clear causal relationships between molecular dysregulation and impaired organism function [7,17].

### 2.1. Spatial and Temporal Control Systems

The basis of disease modeling using *Drosophila melanogaster* is the GAL4/UAS binary expression system, which allows for tissue-specific expression of human disease-related proteins and targeted RNA interference [21,22]. However, relying solely on spatial control often leads to developmental abnormalities that confuse adult-onset conditions [23]. A temporal control system is essential to distinguish between developmental roles and progressive disease mechanisms [24]. The temporal-local gene expression targeting (TARGET) system utilizes a temperature-sensitive GAL80 repressor to limit GAL4 activity until adulthood by varying the rearing temperature from 18 °C to 29 °C [24]. While this temperature change is widely used, it itself can induce an independent heat stress response, potentially altering basal metabolic rate and neurodegeneration rate, making it a significant confounding factor [25,26]. Alternatively, the Gene-Switch system allows chemical induction using hormone analogs such as RU486, which avoids heat stress but requires careful adjustment of drug doses to prevent chemical toxicity [27]. Maintaining a proper balance between these spatial and temporal systems is crucial to ensuring that the observed phenotypes reflect the true pathology of the disease, rather than experimental stress.

Beyond GAL4/UAS, the use of orthogonal binary expression systems such as the Q system (QF/QUAS) and LexA/LexAop allows for the simultaneous and independent labeling or manipulation of multiple tissues within the same individual, facilitating the design of more complex experiments [28]. Cell- or tissue-specific genetic rescue, which selectively reintroduces wild-type transgenes into specific tissues to reverse mutant phenotypes, is the gold standard approach for establishing causal-to-developing tissue relationships and provides particularly valuable information for validating therapeutic target candidates before application to mammalian systems.

### 2.2. Clonal and Mosaic Approaches for Cellular Heterogeneity

While it is possible to manipulate entire organs using tissue-specific driving factors, human pathologies such as cancer and localized tissue degeneration typically arise from a single mutant cell surrounded by normal tissue [29]. To model this cellular heterogeneity, *Drosophila* researchers utilize clonal and mosaic systems, particularly the FLP/FRT system and the mosaic analysis (MARCM) system using suppressive cell markers [30]. These tools make it possible to generate positively labeled homozygous mutant clones within otherwise heterozygous and phenotypically normal tissue [31,32].

This approach is highly effective for distinguishing between cell autonomous effects, which are restricted to the mutant cells themselves, and non-autonomous effects, which alter the behavior of neighboring wild type cells or systemic physiology [33]. In cancer models, mosaic systems have revealed how oncogenic clones actively compete with and eliminate adjacent normal cells through cell competition, and how they secrete systemic factors that drive muscle wasting and metabolic remodeling [34,35].

However, the mosaic method has its own inherent experimental challenges [33]. The size, frequency, and spatial distribution of genetic clones can vary between individuals, which increases data variability and makes quantitative comparison difficult [33,36]. To ensure reproducible clone induction, it is necessary to maintain strict standardization of heat shock duration and driver activity in repeated experiments [33].

### 2.3. Integrated Phenotyping Platforms and Quantitative Readouts

To assess the functional impact of genetic disease models, a comprehensive panel of standardized phenotypic measures is needed [7,37]. *Drosophila* studies excel at high-throughput tracking of overall health indicators such as viability, baseline lifespan, and healthy lifespan indices [38,39]. Behavioral assays, particularly negative gravitaxis assays that measure the innate climbing reflex, are widely used due to their scalability and ease of quantification [40,41]. Automated video tracking systems further extend these capabilities, enabling high-resolution, continuous monitoring of subtle changes in *Drosophila* sleep structure, circadian rhythms, and movement patterns throughout their lifespan [42,43].

However, utmost caution is required when interpreting these macro-level behavioral indicators [44]. Reduced climbing ability and altered sleep patterns are often nonspecific and may reflect a complex interplay of pathologies originating from multiple different organ systems [45,46]. For example, motor dysfunction can result from primary neurodegeneration [47], structural deterioration of muscle tissue [48], or systemic energy depletion due to metabolic stress [49]. Therefore, relying solely on behavioral indicators can lead to misinterpretations of cell-autonomous gene function [44,47,48,49]. To overcome this limitation, macro-behavioral profiling needs to be integrated with organ-level and cellular-level measurements, such as histopathology to confirm cell loss [7], biochemical assays to quantify lipids and carbohydrates [50], and transcriptome profiling to map downstream signaling cascades [51].

Still, identifying which of these organ-level contributions plays a causal role in specific behavioral phenotypes requires manipulation tools with appropriate temporal resolution. Optogenetic tools such as CsChrimson and GtACR offer new strategies for unraveling causal relationships by enabling the precise and rapid activation or deactivation of genetically identified neurons and circuits in time. These complement constitutive or long-term genetic manipulations, allowing researchers to more directly link circuit-level activity with specific behavioral phenotypes [52].

## 3. Strategic Disease Domains in *Drosophila* Research

**Figure 1:**
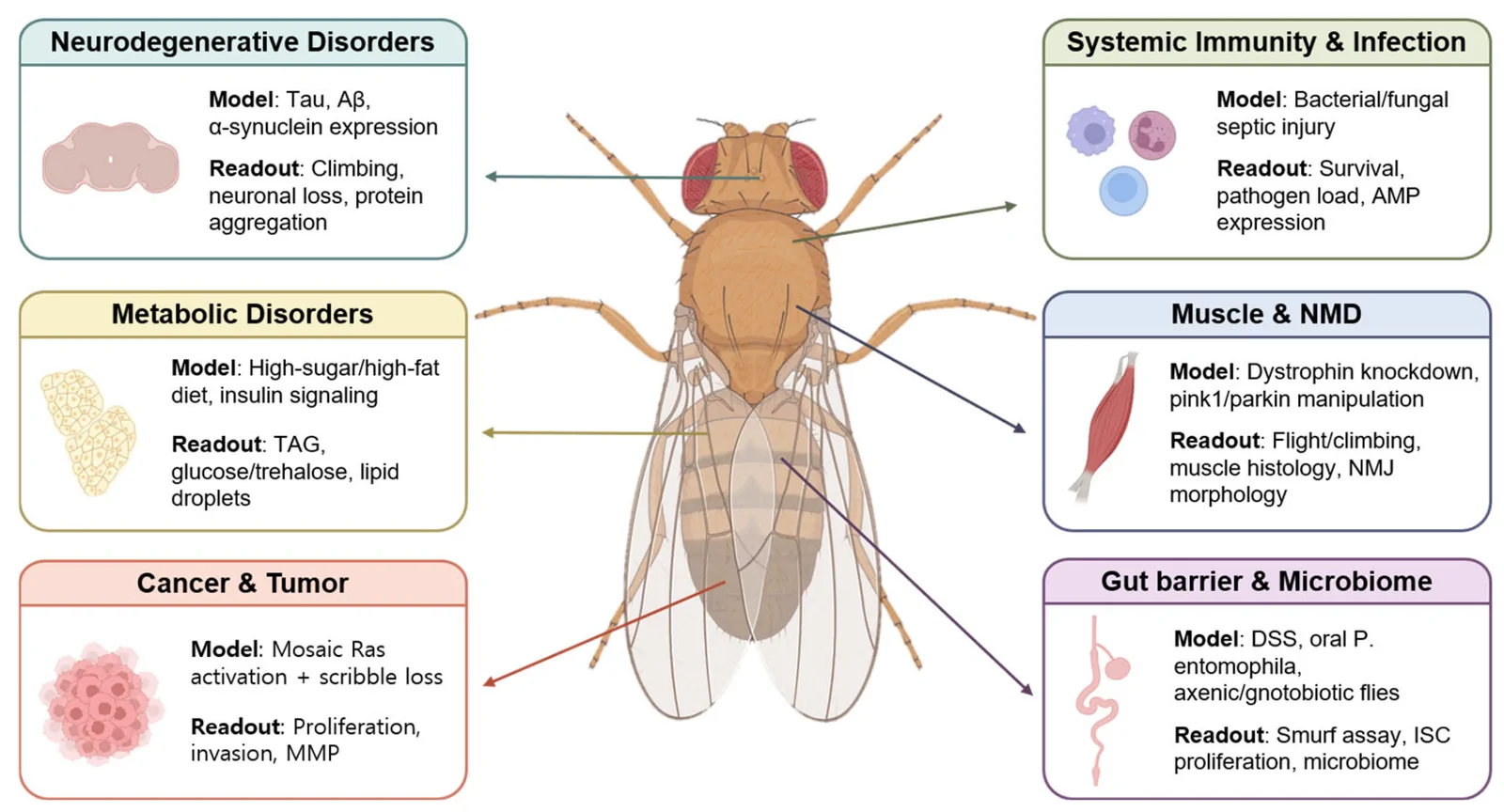
Overview of *Drosophila* Disease Models. Representative modeling strategies and experimental evaluation indicators are shown for neurodegenerative and nervous system diseases, metabolic diseases, cancer, systemic immunity and infections, intestinal mucosal immunity, gut microbiome, and muscle and neuromuscular diseases. TAG: Triacylglycerol; MMP: Matrix metalloproteinase; DSS: Dextran sulfate sodium; ISC: Intestinal stem cells; NMJ: Neuromuscular junction.

The application of the spatial, temporal, and clonal genetic toolkits and phenotypic analysis platforms to specific biomedical fields has resulted in the establishment of diverse disease models that capture the essential characteristics of human pathology [53,54]. Rather than attempting to replicate the anatomical complexity of vertebrate organs, these models identify core pathogenesis axes such as disruption of protein homeostasis [55], metabolic abnormalities [56], and breakdown of tissue barriers [57]. By focusing on conserved molecular pathways, researchers can elucidate how primary genetic defects lead to progressive cellular and organismal-level functional decline [58]. The following sections examine six major disease areas in which *Drosophila* research has made significant contributions to elucidating mechanisms, and identify common modeling strategies, to avoid ensuring translational relevance (Table 1 and Figure 1) [59,60].

### 3.1. Neurodegenerative and Nervous System Disorders

Neurodegenerative diseases are characterized by a set of common etiological features, including disruption of proteostasis, toxic accumulation of misfolded proteins, and progressive synaptic loss [77]. To investigate these processes, *Drosophila* models typically employ techniques to directly and indirectly express human disease-related proteins, such as amyloid-beta and tau in Alzheimer’s disease and alpha-synuclein in Parkinson’s disease, within whole neurons or cell-type-specific strains [78,79]. These models effectively replicate key features of human neurodegeneration, including progressive motor impairment, shortened lifespan, and structural neuronal loss [80]. Standardized quantification of these phenotypes relies heavily on negative geomagnetic tactic assays to track age-related decline in motor function, in conjunction with histological analyses of fly brains to measure vacuolar lesions and protein aggregation dynamics [81].

However, a major experimental pitfall in these paradigms is their reliance on the overexpression of elevated levels of non-physiological genes [82]. Expressing enormous amounts of toxic human proteins via strong promoters often compresses decades of human disease processes into the 50-day lifespan of *Drosophila*, frequently resulting in nonspecific cellular stress and anthropogenic toxicity that do not accurately reflect the gradual mechanisms of human aging [82,83]. To ensure translational validity, researchers must carefully control gene expression levels, utilize low-expression or inducible drivers, and conduct parallel loss-of-function studies on endogenous *Drosophila* orthologues to distinguish between general cellular overload and specific pathogenic pathways [84].

In addition to neurodegenerative diseases that develop in adulthood, *Drosophila* have also been used as a model for neurodevelopmental disorders. In neurodevelopmental disorders, genetic abnormalities do not cause progressive functional decline in adulthood but rather disrupt the formation of neural circuits during development. For example, fruit fly homologs of genes associated with human autism and intellectual disability, such as those located in the 16p11.2 deletion region, have been used to elucidate how gene dose imbalances during development cause synaptic and behavioral phenotypes associated with human neurodevelopmental disorders [37].

### 3.2. Metabolic Disorders

Metabolic disorders such as obesity, type 2 diabetes, and fatty liver reflect a complex interplay of nutrient overload, insulin resistance, and ectopic lipid accumulation. *Drosophila* provides an effective platform for elucidating these pathologies because insulin receptor signaling and nutrient sensing mechanisms are conserved at the pathway level [65]. Researchers typically model metabolic stress by exposing *Drosophila* to high-carbohydrate or high-fat diets [67] or by genetically disrupting key signaling components such as the insulin receptor and its downstream substrate, *chico* [85]. The functional outcomes of these models are quantified using standardized biochemical indicators, including whole-body triglyceride measurements, hemolymph glucose and trehalose measurements, and histological staining of lipid droplets in the adipocyte to assess tissue-specific lipid accumulation [86].

However, a major experimental hardship in *Drosophila* metabolic studies is the lack of standardization of baseline culture medium formulations across research institutions [87]. The exact composition of standard *Drosophila* diets varies from laboratory to laboratory, particularly in terms of the raw materials and concentrations of yeast, cornmeal, and nutritional additives. These baseline dietary factors deeply influence nutrient sensing, life history traits, and gut microbiota composition, so a high-carbohydrate diet that induces significant insulin resistance in one institution may cause only mild or compensatory metabolic changes in another [88]. To improve comparability between studies, it is essential that researchers meticulously report the precise quantitative breakdown of dietary components and establish a standardized reference diet for metabolic phenotypic analysis [87,89].

### 3.3. Cancer and Tumor

Cancer biology in *Drosophila* leverages the organism’s powerful cloning tools to model genetic coordination, tissue structure loss, and systemic tumor-host interactions [90,91]. Modern *Drosophila* tumor models replicate the multi-step nature of human cancer development by co-activating oncogenic signaling pathways in parallel with disruption of tumor suppressors and cell polarity genes, rather than relying on simple single-gene mutations [90]. A typical approach involves generating mosaic clones that express active *Ras* while simultaneously carrying mutations in the cell polarity gene scribble [90]. These synergistic clones exhibit key features of human malignancies, including uncontrolled proliferation, tissue invasion, and induction of matrix metalloproteinases [90,91]. Furthermore, these localized tumors release systemic signaling molecules that induce cachexia-like wasting syndrome, characterized by the breakdown of host muscle and fat tissue [92].

But the *Drosophila* cancer model has a significant interpretive limitation due to its high clonal homogeneity [93]. Human tumors are highly heterogeneous ecosystems with diverse secondary mutations and constantly evolving subclones, whereas a typical *Drosophila* mosaic system forms a genetically identical clonal population [94]. This genetic homogeneity makes it difficult to model inter-clonal competition within tumors, treatment resistance caused by existing sub-clonal diversity, or the complex clonal evolutionary trajectories observed during the clinical course of humans [91,93]. Therefore, researchers should view the *Drosophila* platform not as a complete simulation of the human tumor ecosystem, but primarily as a tool to elucidate basic signaling networks and early oncogenic synergies [91].

### 3.4. Systemic Immunity and Infectious Diseases

*Drosophila* systemic immunity relies entirely on a highly sophisticated innate immune system that shares deep evolutionary roots with human innate defense mechanisms [94,95]. The *Drosophila* systemic immune response is controlled by two major signaling cascades: the Toll pathway, which recognizes fungal and Gram-positive pathogens, and the Immunodeficiency (Imd) pathway, which responds to Gram-negative bacteria [94]. Researchers typically replicate systemic infections using septic injury as a model, injecting bacterial and fungal pathogens directly into the hemolymph at precise doses using microinjection needles [96]. The immune response is quantified through viability, bacterial clearance assays, and measurements of transcriptional induction of downstream antimicrobial peptides produced by fat bodies and circulating blood cells [94,96].

However, the complete absence of an adaptive immune system remains a fundamental limitation in interpreting the *Drosophila* infection and inflammation model [97]. Because *Drosophila* lacks somatic recombination mechanisms, functional lymphocytes, and antibody-mediated immunological memory, it cannot replicate chronic adaptive immune diseases, vaccine-mediated defense mechanisms, or complex autoimmune diseases caused by T-cell and B-cell dysregulation [97]. While *Drosophila* is an excellent tool for studying immediate and immobilized innate immune responses to pathogens, researchers must exercise caution when generalizing these findings to chronic, multilayered mammalian inflammatory diseases in which adaptive immunity plays a dominant role [94,97].

### 3.5. Gut Mucosal Immunity, Barrier Function, and Microbiome

The midgut of the fruit fly serves as a powerful model of gut biology, capturing the integrity of the epithelial barrier, local mucosal immunity, and the complex interactions between the commensal microbiota [72]. Pathological damage in this region often manifests chronic epithelial damage, hyperactivation of intestinal stem cells leading to hyperproliferation, and breakdown of the chemical and physical barriers separating the intestinal lumen from the hemolymph [72,98]. Researchers routinely model the paradigm of human inflammatory bowel disease by orally administering chemical irritants such as dextran sulfate sodium, introducing intestinal pathogens such as *Pseudomonas entomophila*, or raising germ-free flies to assess the absence of the microbiome [72,98]. Intestinal permeability is traditionally measured using the Smurf assay, in which flies are administered an unabsorbable blue dye, and the leakage of the dye into the body cavity is used as a direct indicator of barrier dysfunction [99]. These measurement results are used in combination with immunostaining of mitotic markers to quantify the division rate of stem cells and high-throughput sequencing to monitor changes in microbial composition [72,99].

A major experimental challenge in studies of the fruit fly gut is the significant inter-institutional variability in the composition of the baseline microbiome [100]. Because fruit flies acquire their microbiome from their rearing environment, the specific bacterial genera residing in the gut can vary between institutions, and even between different incubators within the same building [100,101]. This environmental variability directly impacts baseline immune status, stem cell turnover rates, and susceptibility to chemical and infectious stress, frequently leading to a lack of reproducibility across different laboratories [100,101]. To mitigate this significant pitfall, research in this area requires the description of strict rearing conditions, the use of communal rearing or shared bedding to homogenize the microbiome across experimental cohorts, or the introduction of defined gnotobiotic models in which known bacterial strains are introduced into germ-free flies [101].

### 3.6. Muscle and Neuromuscular Disorders

*Drosophila* muscle tissue, particularly indirect flight muscle, and the neuromuscular junctions that innervate it, provides a readily available in vivo model for studying muscle diseases, dystrophy, and motor neuron diseases [102,103]. Major etiologies in these diseases include disruption of sarcomere structure within myofibrils, impaired excitation-contraction coupling, calcium dysregulation, and impaired mitochondrial energy production [102]. Common modeling strategies include muscle-specific knockdowns of conserved structural orthologues such as the *Drosophila dystrophin* gene, or disruption of mitochondrial quality control factors such as *Pink1* and *Parkin* [102,103]. Quantitative assessment of muscle function is performed using physiological indicators such as flight ability tests, negative gravitaxis assays, histological sequence scoring of polarized muscle fibers, and morphological quantification of synaptic buttons at larval neuromuscular junctions [104].

A major interpretive challenge in these models is the difficulty in distinguishing between primary muscle degeneration and upstream neuronal dysfunction [105]. Because coordinated movement integrates input from both the central nervous system and the peripheral muscular system, severe climbing and escape behavior impairments caused by subtle defects in motor neuron firing or synaptic transmission can easily be misinterpreted as being due to primary muscle disease [106]. To establish true tissue-specific causality, researchers must avoid overinterpreting the overall behavioral phenotype alone. It is crucial to conduct parallel gene rescue experiments using independent pan neuronal and muscle-specific drivers to confirm that the phenotype is selectively reproduced or restored only when genetic manipulation is limited to the specific tissue being targeted [107,108].

## 4. Cross-Disease Convergence Frameworks

**Figure 2:**
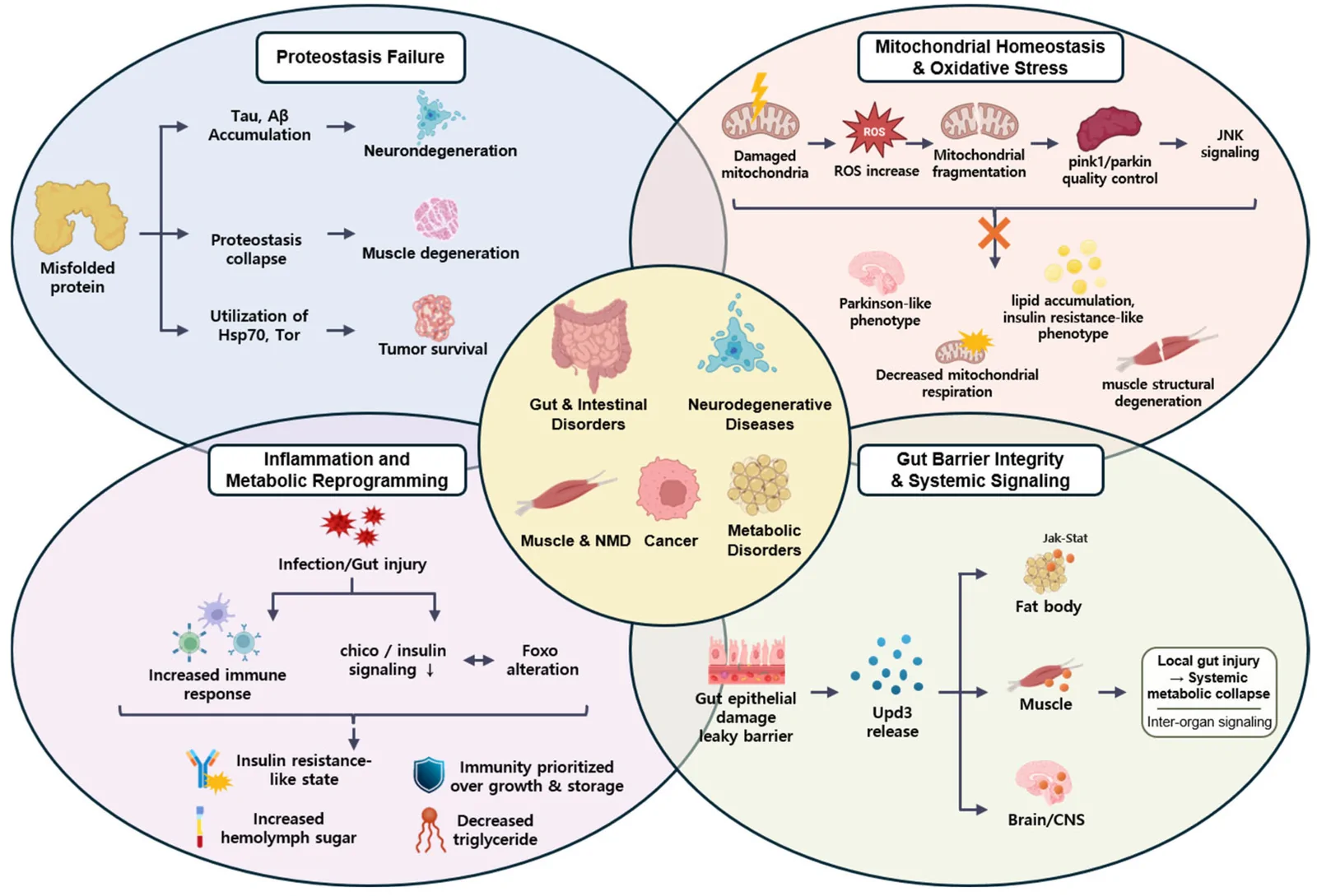
Cross-Disease Convergence of Conserved Signaling Networks in *Drosophila* melanogaster. The figure shows four major molecular hubs where phenotypic disease regions (neurodegenerative diseases, muscle and neuromuscular diseases (NMD), cancer, metabolic diseases, and gastrointestinal diseases). ROS: Reactive oxygen species.

One major advantage of modeling diverse pathologies in *Drosophila* is the ability to uncover shared molecular hubs that unify phenotypically distinct disorders (Figure 2) [109]. While clinical classifications often separate neurodegeneration, metabolic decline, and cancer into isolated medical specialties, in vivo genetic screening in the fly reveals that these conditions frequently exploit the same conserved signaling networks [109,110]. By leveraging tissue specific and temporally controlled genetics, researchers can determine whether these shared pathways represent passive downstream consequences of cellular injury or active, causal drivers of systemic decline [109]. The following sections explore four major mechanical axes where diverse human diseases converge within the *Drosophila* platform, demonstrating how core cellular stress responses, mitochondrial quality control, chronic inflammation, and inter-organ communication function as universal modifiers of organismal health [110].

One of the major advantages of using *Drosophila* to model diverse diseases is the ability to elucidate common molecular hubs that link phenotypic diseases (Figure 2) [109]. Clinical classifications often categorize neurodegeneration, metabolic dysfunction, and cancer as separate specialties, but in vivo genetic screening using *Drosophila* has revealed that these diseases often utilize the same conserved signaling networks [109,110]. By employing tissue-specific and time-controlled genetic techniques, researchers can determine whether these common pathways are passive downstream consequences of cellular damage or active causative factors of systemic functional decline [109]. The following sections explore four key mechanistic axes through which diverse human diseases converge on the *Drosophila* platform, revealing how core cellular stress responses, mitochondrial quality control, chronic inflammation, and inter-organ communication act as universal modifiers of individual health [110].

### 4.1. Common Mechanisms Between Protein Homeostasis Disruption and Diseases

Disruption of proteostasis and accumulation of misfolded proteins are prominent features of tissue-specific diseases [110]. In *Drosophila* neurodegeneration models, toxic aggregation of human tau protein and amyloid-beta overloads the ubiquitin-proteasome system, inducing endoplasmic reticulum stress and activating a conserved unfolded protein response [111]. Importantly, this same proteostatic breakdown is also observed in muscle structure breakdown models such as *Dys* mutants, in which the intracellular chaperone network is destabilized by mechanical stress [112]. Genetic studies in *Drosophila* have demonstrated that overexpression of certain molecular chaperones, such as the heat shock protein Hsp70, can simultaneously restore motor dysfunction in Alzheimer’s disease models and muscle fiber degeneration in muscular dystrophy models, providing direct causal evidence for the convergence of these conditions [113]. Conversely, while neurodegenerative and muscular diseases are exacerbated by prosteostatic dysfunction, malignant tumor clones often upregulate the same protective chaperone mechanisms and Tor signaling cascades to suppress stress-induced apoptosis, enabling the survival and proliferation of abnormal cells [114]. In these diverse models, manipulating Tor-regulated autophagy and *Xbp1*, a transducer of the unfolded protein response, has shown that these networks not only label damaged tissue but also function as bidirectional upstream that determine cell survival or death across unrelated disease regions [115]. These proteostasis-focused strategies run parallel to efforts to develop chaperone-induction therapies and proteostasis-modulating therapies for human neurodegenerative and muscular diseases.

### 4.2. Mitochondrial Homeostasis and Oxidative Stress

Mitochondrial dysfunction and the resulting accumulation of reactive oxygen species represent another major convergence point where tissue-specific pathologies overlap [116]. In the context of neuromuscular diseases, failure of mitochondrial quality control is linked to cell death. *Drosophila* models have played a crucial role in elucidating the causal genetic mechanisms of this process through studies of the *Pink1* and *Parkin* pathways. Mutations in these genes cause severe mitochondrial fragmentation, leading to loss of dopaminergic neurons in Parkinson’s disease models and significant structural deterioration in indirect flight muscle [73,117].

Importantly, the effects of this pathway extend beyond neuromuscular tissue to metabolic homeostasis. Upregulation of *Pink1* and *Parkin* not only slows neurodegeneration but also improves mitochondrial respiration in the fat body, suppresses ectopic lipid accumulation, and alleviates insulin resistance-like states induced by high-carbohydrate diets [118]. This systemic restorative effect indicates that mitochondrial quality control is not a requirement specific to nerve cells, but a universal determinant of tissue health [118]. Therefore, this pathway is directly relevant to human disease, as mutations in *pink1* and *parkin* are a well-established cause of autosomal recessive Parkinson’s disease in humans.

Furthermore, oxidative stress caused by dysfunctional mitochondria often triggers a conserved JNK signaling cascade. In degenerated neurons and muscles, JNK activation promotes apoptosis, while in tumor models, oxidative stress-mediated JNK signaling can promote cell survival and invasion [119,120]. By positioning Pink1, Parkin, and JNK at the crossroads of these diseases, *Drosophila* studies highlight how targeting a single mitochondrial quality control axis can modulate different degenerative and proliferative pathologies across the organism [121].

### 4.3. Inflammation and Metabolic Reprogramming

The bidirectional relationship between chronic innate immune activation and systemic metabolic rewiring forms a fundamental axis in disease resolution [122]. In *Drosophila*, systemic infections and local intestinal barrier disruption not only trigger isolated immune responses but also fundamentally reprogram the host’s carbohydrate and lipid metabolism [122]. Chronic activation of the Toll and Imd pathways by pathogens or epithelial damage promotes nuclear translocation of NF-κB orthologues such as Dif and Relish, not only in circulating blood cells but also extensively in the adipocyte [123].

Importantly, this sustained immune activation directly interferes with the normal nutrient signaling cascade [122,124]. This enhancement of inflammatory signaling suppresses the function of Chico, an insulin receptor substrate in *Drosophila*, thereby inducing a systemic insulin resistance state [122]. Simultaneously, chronic inflammation modulates the activity of the conserved metabolic transcription factor Foxo, shifting energy resources from growth and lipid storage to immediate immune defense [125]. This forced metabolic reprogramming leads to hyperglycemic-like increases in hemolymphatic glucose levels and rapid depletion of triglyceride stores in the fat body, providing clear genetic evidence that the inflammatory tone acts as a direct upstream regulator of systemic metabolic homeostasis [126]. This link mirrors the well-documented association between chronic inflammation and insulin resistance in human metabolic syndrome and type 2 diabetes.

### 4.4. Gut Barrier Integrity and Systemic Signaling

The physiological effects of localized tissue damage are limited to a single organ, and *Drosophila* melanogaster is attracting attention as a major model for elucidating these distant inter-organ communication networks [127]. This systemic interaction is demonstrated after the breakdown of the gut barrier function [127]. When the midgut epithelium is damaged by chemical stress, chronic inflammation, or tumor growth, the damaged intestinal cells respond by secreting systemic signaling molecules into the body fluids [128]. The main mediator of this response is Unpaired 3 (upd3), a cytokine that is orthologue to mammalian interleukin-6 [128,129]. Once released, upd3 acts as a long-range endocrine signaler, traveling through the circulatory system and binding on receptors to distant target tissues, including the fat body and the central nervous system [128,129].

This systemic circulation of upd3 triggers potent activation of the conserved Jak-Stat signaling cascade in receptor organs [130]. In fat bodies and muscle tissue, sustained Jak-Stat signaling suppresses anabolic pathways and promotes the mobilization of stored energy reserves, leading to a phenotype of marked muscle and tissue atrophy closely resembling human cancer cachexia [130,131]. Simultaneously, gut-derived upd3 acts on neural circuits in the brain, altering feeding behavior and metabolic output [132]. By elucidating these endocrine-like networks, *Drosophila* studies provide conclusive genetic evidence that local mucosal barrier dysfunction is a major upstream trigger for overall metabolic collapse and systemic organ dysfunction [127,132]. This gut-to-muscle signaling axis closely resembles the IL-6/JAK-STAT-driven wasting observed in human cancer cachexia.

## 5. Translational Pipelines and Reproducibility

The ultimate utility of *Drosophila* disease modeling lies in its ability to accelerate translational pipelines and provide a reproducible experimental platform. Rather than functioning in isolation, the *Drosophila* platform acts as an efficient screening mechanism within the broader biomedical research ecosystem, identifying high-priority therapeutic targets and lead compounds before conducting costly mammalian trials [133,134]. However, bridging the evolutionary gap between *Drosophila* and human clinical outcomes requires both rigorous screening methods and strict adherence to reproducibility criteria [135]. This section outlines the strategic integration of *Drosophila* models into preclinical drug discovery and establishes a methodological framework for addressing confounding variables that often impair interlaboratory reproducibility [133,135].

### 5.1. Phenotypic Drug Screening and Translational Pipelines

*Drosophila* melanogaster provides an excellent in vivo model for high throughput chemogenetic and phenotypic drug screening [136,137]. Unlike cell culture assays that lack physiological complexity and mammalian models that are limited by low throughput and high cost, screening at the individual *Drosophila* level allows for the simultaneous assessment of systemic drug absorption, metabolic conversion by the fat body, and toxicity to target organs [136]. Researchers are leveraging these advantages by using automated dispensing systems to place larvae and adults in multi-well plates containing chemically diverse compound libraries [138]. Phenotypic measurements, such as recovery of lethal phenotypes, recovery of motility, or reduction in tissue-specific cell death, enable unbiased identification of bioactive compounds [136,137].

Furthermore, these screenings do not require prior knowledge of specific molecular targets, thus routinely revealing unexpected chemical mechanisms [137]. Promising lead compounds identified in *Drosophila* serve as validated starting points for chemical optimization and subsequent validation in mouse models and patient-derived organoids [139]. This stepwise pipeline significantly reduces preclinical failure rates by ensuring that only compounds with demonstrated efficacy in vivo and an acceptable safety profile proceed to vertebrate studies [136,139].

### 5.2. Methodological Robustness and Reproducibility Checklists

Despite the clear advantages of using *Drosophila* as a research platform, the biomedical community often heavily criticizes invertebrate studies due to phenotypic drift and a lack of inter-laboratory reproducibility [140]. To establish *Drosophila* as the gold standard for translational research, researchers must implement standardized reproducibility checklists that consider important confounding variables [140,141]. First, controlling the genetic background is crucial, as variability in modified loci between strains from different laboratories can completely mask or amplify disease phenotypes [141,142]. Researchers should strictly backcross experimental strains to a co-isogenic reference background, such as a standard w1118 strain, to eliminate background-induced noise [142].

Secondly, environmental parameters must be strictly controlled and reported with transparency [140,143]. Factors such as stocking density, which directly affect larval nutritional status and adult body size, and the precise quantitative breakdown of baseline culture medium components must be controlled across all replicate groups in the experiment [143]. Finally, phenotypic analysis with respect to aging requires temporal consistency, as even slight fluctuations in incubator humidity and temperature over time can alter the timing of the onset of progressive neurodegeneration and metabolic decline [140,144]. Adherence to these standardized reporting criteria is essential to transforming *Drosophila* research findings into robust and universally verifiable biomedical knowledge [140,141]. Collectively, the advantages and disadvantages discussed throughout the preceding sections are summarized in Table 2.

## 6. Conclusions

The strategic use of *Drosophila melanogaster* in human disease modeling represents a paradigm shift in how researchers unravel complex biomedical phenomena. The fundamental usefulness of this *Drosophila* platform lies not in replacing vertebrate physiological functions, but in its role as intellectual compass guiding mammalian research. By removing the high-dimensional physiological noise inherent in mammalian models, *Drosophila* studies identify core molecular mechanisms and establish remarkably clear and decisive causal relationships. Insights gained from these simplified in vivo systems provide a focused framework that maximizes the efficiency and success rate of subsequent translational studies in human tissues and animal models.

The new frontiers of disease modeling using *Drosophila* will lie at the intersection of classical genetics and cutting-edge technology. The so-called “AI-omics” framework, which integrates artificial intelligence (AI) and machine learning with high-dimensional multi-omics datasets, will enable predictive modeling of complex gene-environment interactions and systemic inter-organ networks. Simultaneously, the increased use of automated, high-throughput phenotyping platforms will solidify *Drosophila*’s position as an indispensable tool for personalized medicine and rapid functional validation of rare human mutations. As biomedical research shifts towards a system-level understanding, the conceptual clarity offered by the biological structure of *Drosophila* will remain essential for translating vast genomic datasets into the discovery of actionable therapies.

## Figures and Tables

**Table 1:** Overview of *Drosophila* disease models and key experimental features.

Disease Category	Related Disease	Method	Target Tissues	Core Phenotypes	Ref.
Neurodegenerative diseases	Alzheimer’s disease	Pan-neuronal Aβ42 expression (GAL4/UAS)	CNS	Aβ42 accumulation, learning/memory impairment, age-dependent neurodegeneration	[61]
	Alzheimer’s disease	Neuronal Tau expression (elav-GAL4)	CNS	Tau accumulation/phosphorylation, brain vacuolization, neuronal loss	[62]
	Parkinson’s disease	αSynA53T expression (TH-GAL4/elav-GAL4)	Nervous	Dopaminergic neuronal loss, α-synuclein accumulation, locomotor impairment	[63]
Metabolic diseases	Type 2 diabetes and obesity	High-sucrose diet	Fat body	Hyperglycemia, insulin resistance, TAG accumulation	[64]
	Obesity	High-fat diet feeding	Fat body	TAG accumulation, obesity, impaired cardiac contraction/rhythm	[65]
	Impaired insulin signaling	InR hypomorphic allele combination	Fat body	Reduced insulin signaling, impaired glucose homeostasis	[66]
	Impaired insulin signaling	*Chico* (P-element/mutagenesis)	Fat body	Reduced insulin signaling, increased body fat	[67]
	Fatty liver	Pvr–PI3K–Akt–TOR RNAi	Muscle, fat body	Lipid accumulation in oenocytes/fat body, increased whole-body TAG	[68]
Cancer	Malignant tumors	MARCM scrib−/− clones + RasV12 expression	Eye-antennal imaginal disc	Tumor clone overgrowth, tissue invasion, basement membrane degradation	[69]
Infectious diseases	Fungal and Gram-positive bacterial infection	Natural/septic infection	Fat body, hemolymph	Reduced antifungal response, increased fungal proliferation, decreased survival	[70]
	Gram-negative bacterial infection	Septic injury	Fat body, hemolymph	Reduced AMP response, impaired bacterial clearance, increased mortality	[71]
Gut-related diseases	Inflammatory intestinal injury	Oral DSS/bleomycin administration	Midgut	Epithelial damage, ISC hyperproliferation, increased intestinal permeability	[72]
	Enteric bacterial infection	Oral *P. entomophila* feeding	Midgut	Intestinal epithelial destruction, bacterial proliferation, infection-associated mortality	[73]
	Microbiota depletion and defined microbial colonization	Egg sterilization and bacterial inoculation	Lumen	Germ-free status or selective colonization by defined strains	[74]
Muscular diseases	Muscular dystrophy	Muscle *Dystrophin* RNAi	Body wall, muscles	Progressive muscle degeneration, myofibrillar disruption, locomotor impairment	[75]
	Mitochondrial muscle degeneration	*pink1* deletion	Indirect flight muscles	Mitochondrial damage, flight muscle degeneration, impaired flight/climbing	[76]
	Mitochondrial muscle degeneration	P-element excision/EMS	Indirect flight muscles	Mitochondrial abnormalities, apoptotic muscle degeneration, impaired flight/climbing	[48]

**Table 2:** Summary of the Advantages and Limitations of *Drosophila melanogaster* as a Model for Human Disease.

Feature	Advantages	Disadvantages/Limitations
Genetic tractability	Enormous collection of genetic tools (GAL4/UAS, RNAi, CRISPR/Cas9, MARCM) enabling precise spatial and temporal control	Genetic redundancy and background effects can mask or amplify phenotypes; require careful control lines
Speed and cost	Short generation time (~10 days), low husbandry cost, large sample sizes for statistical power	Compressed lifespan can force non-physiological, high-level transgene expression
Conserved pathways	~75% of human disease genes have functional orthologs; core signaling pathways highly conserved	Anatomical simplicity precludes modeling of complex organs (e.g., multi-chambered heart, kidney)
Immunity and physiology	Well-characterized innate immune system (Toll/Imd); accessible fat body, gut, and neuromuscular systems	No adaptive immune system; cannot model antibody-mediated or T/B-cell-driven disease
Translational pipeline	High-throughput in vivo drug and genetic screening platform prior to costly mammalian trials	Evolutionary distance from humans limits direct extrapolation of pharmacokinetics and toxicology

## Data Availability

No new data were created or analyzed in this study. Data sharing is not applicable to this article.
